# Overcoming translational and manufacturing barriers of CAR-T cell therapy in the Brazilian landscape

**DOI:** 10.1016/j.omton.2026.201363

**Published:** 2026-10-14

**Authors:** Cleyson da Cruz Oliveira Barros, Larissa Cardoso Zanetti, Lays Adrianne M. Trajano-Silva, Julia Carnaz Benincasa, Erica Kássia Sousa Vidal, Ingrid Ferreira de Souza, Robledo Aires Ferreira, Lilian de Oliveira Coser, Leonardo Javier Arcuri, Jamile Ramos da Silva, Yasmin Amirato, Rodrigo Coelho Ventura Pinto, Augusto Barbosa Junior, Juliana Aparecida Preto de Godoy, Raquel de Melo Alves Paiva, Oswaldo Keith Okamoto, Nelson Hamerschlak, Lucila Nassif Kerbauy

**Affiliations:** 1 Hospital Israelita Albert Einstein, Cell Therapy Center, Sao Paulo, Brazil; 2 Universidade de São Paulo, Genetics and Evolutionary Biology, Sao Paulo, Brazil; 3 Hospital Israelita Albert Einstein, Academic Research Organization, Sao Paulo, Brazil; 4 FIOCRUZ, Institute of Technology on Immunobiologicals (Bio-Manguinhos), Rio de Janeiro, Brazil

**Keywords:** CAR-T cell therapy, cell manufacturing, academic development, Brazil, hematologic malignancies

## Abstract

Chimeric antigen receptor (CAR) T cell therapy represents an advancement in the treatment of hematologic malignancies, offering remissions in relapsed or refractory disease. However, its global implementation is limited by high production costs, logistical complexity, and the need for specialized facilities that comply with biosafety and regulatory standards. In Brazil, all CAR-T products are imported, leading to high costs and delays in patient access. To overcome these barriers, academic institutions have established local CAR-T manufacturing platforms. This review provides an overview of the scientific, technical, and regulatory aspects of academic CAR-T development in Brazil, highlighting key advances and challenges. We discuss cell sources, gene-transfer systems, manufacturing strategies, quality control (QC), and regulatory frameworks. Efforts from institutions such as Hospital Israelita Albert Einstein, University of São Paulo (USP), Butantan Institute, National Cancer Institute (INCA), and Oswaldo Cruz Foundation (Fiocruz) demonstrate complementary approaches. These initiatives underscore the feasibility of developing CAR-T therapies in low- and middle-income countries through innovation, technology transfer, and financing. Finally, we address the integration of CAR-T therapy into the Brazilian public health system, emphasizing workforce training, clinical logistics, and cost-effectiveness to ensure access. Overall, the Brazilian experience illustrates how academic collaboration can translate research into clinical application.

## Graphical abstract

**Figure:**
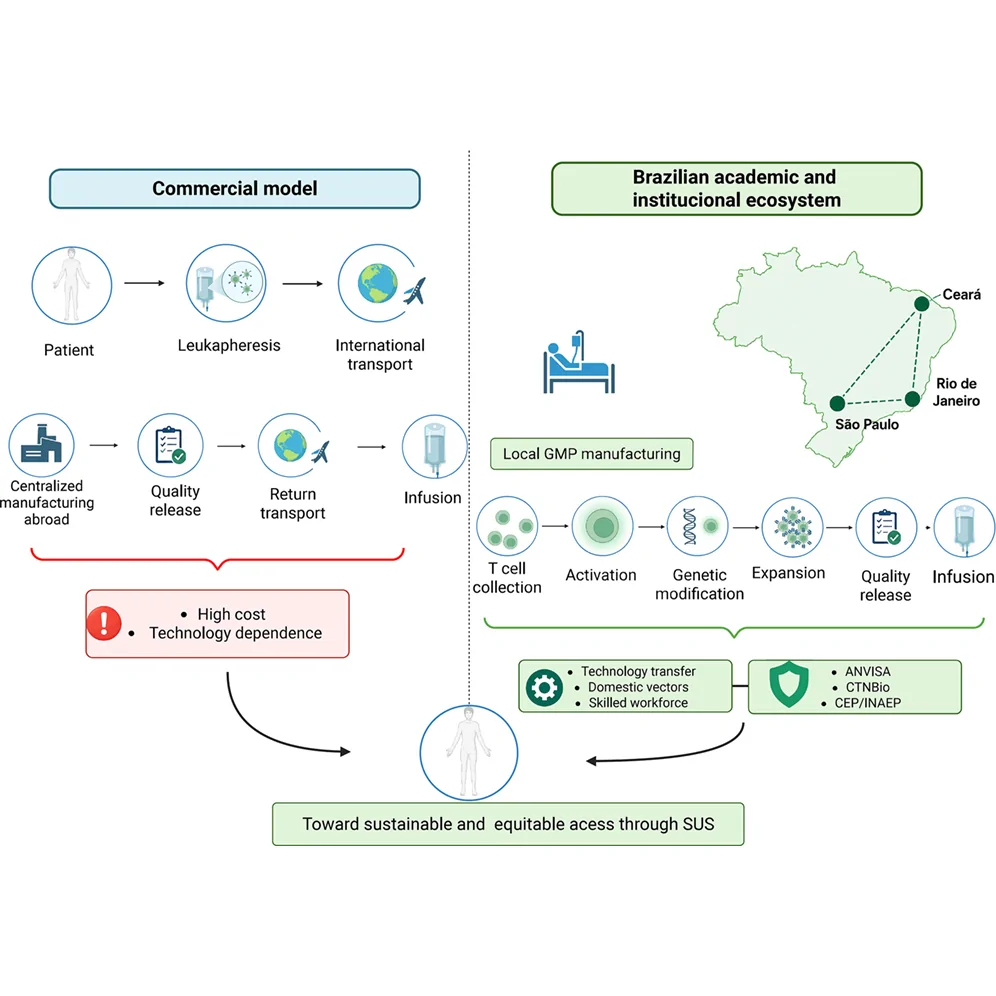

## Teaser

CAR-T therapy has transformed hematologic cancer treatment, yet global access remains highly unequal. Brazil illustrates both the feasibility and challenges of developing locally manufactured CAR-T therapies. This review examines Brazil’s scientific, manufacturing, regulatory, and translational landscape, highlighting technological autonomy, sustainable financing, workforce development, and healthcare integration as essential to achieving equitable access.

## Introduction

In recent years, chimeric antigen receptor (CAR) T cell therapy has emerged as one of the most promising strategies in modern oncology, representing a paradigm shift in the treatment of hematologic malignancies and solid tumors.^1^ By redirecting the cytotoxic activity of T lymphocytes toward specific tumor-associated antigens, CAR-T therapies have demonstrated unprecedented clinical efficacy in patients with relapsed or refractory disease considered incurable.^2^^,^^3^ However, despite these remarkable clinical outcomes, access to CAR-T therapy remains highly unequal worldwide, particularly in low- and middle-income countries, where manufacturing complexity, regulatory requirements, infrastructure limitations, and high treatment costs substantially restrict clinical implementation.

The broad implementation of CAR-T therapies critically depends on the structure of manufacturing, regulatory, and translational workflows. CAR-T products require complex, tightly controlled manufacturing processes, including leukapheresis, cell activation, genetic modification, *ex vivo* expansion, quality control (QC) testing, and timely reinfusion into patients. These requirements impose substantial logistical, infrastructural, and financial challenges that have directly affected global access to CAR-T therapies. Brazil exemplifies many of these challenges. Although commercial CAR-T products have become available in the country, they remain entirely dependent on imported technologies, limiting patient access due to elevated costs, lengthy manufacturing timelines, and limited availability. In response, Brazilian academic institutions and public research centers have established local manufacturing platforms, clinical trials, and technology-development initiatives aimed at building a sustainable national CAR-T ecosystem.

This review aims to provide a comprehensive overview of the scientific, technical, and regulatory aspects of CAR-T cell therapy development in Brazil, with emphasis on the challenges faced by academic and public research institutions.

## The Brazilian CAR-T ecosystem

Unlike high-income countries, where commercial CAR-T products are integrated into established healthcare systems, Brazil still faces substantial barriers related to manufacturing infrastructure, regulatory complexity, technology dependence, workforce training, and financial sustainability. Consequently, the development of a national CAR-T ecosystem has relied primarily on collaborations among universities, public research institutions, hospitals, funding agencies, and regulatory authorities.^4^^,^^5^^,^^6^^,^^7^^,^^8^

Over the last decade, Brazil has progressively established a network of institutions dedicated to developing local CAR-T manufacturing capabilities and expanding access to advanced cellular therapies. Rather than replicating commercial manufacturing models, these initiatives have focused on building academic platforms that integrate research, process development, clinical translation, and technology transfer. This collaborative strategy has enabled the country to develop expertise across multiple stages of the CAR-T value chain, including vector production, preclinical development, good manufacturing practice (GMP) manufacturing, and investigator-initiated clinical trials.^4^^,^^5^^,^^6^^,^^7^^,^^8^^,^^9^^,^^10^

Reference institutions, including Hospital Israelita Albert Einstein, the University of São Paulo (USP), Butantan Institute, National Cancer Institute (INCA), Oswaldo Cruz Foundation (Fiocruz), and Ribeirão Preto Blood Center, have assumed complementary roles within this ecosystem (Figure 1). While Hospital Israelita Albert Einstein has pioneered hospital-based point-of-care manufacturing and investigator-initiated clinical trials,^9^ it is also advancing multiple preclinical and translational initiatives in advanced therapies. USP and Butantan Institute have established independent academic clinical development programs.^11^ Fiocruz has focused on strengthening national technological autonomy through domestic GMP viral vector production,^12^ whereas INCA has expanded translational and preclinical research programs.^13^ Collectively, these initiatives demonstrate that Brazil is progressively developing expertise throughout the CAR-T production pipeline rather than concentrating efforts on a single institution, highlighting the collaborative nature of the national ecosystem.^4^^,^^5^^,^^6^^,^^7^^,^^8^^,^^9^^,^^10^

**Figure 1:**
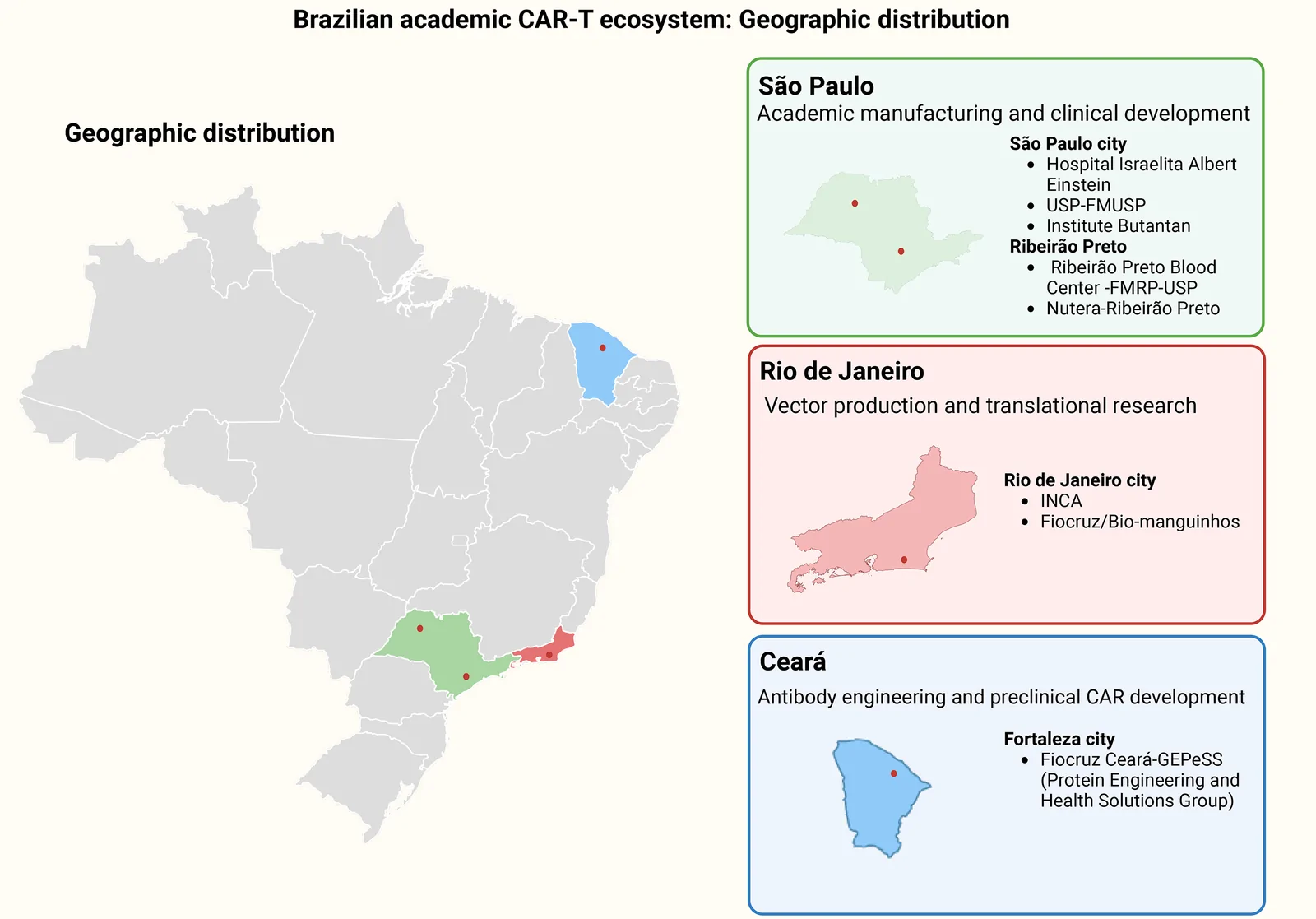
Brazilian academic CAR-T ecosystem: Geographic distribution (Left) Geographic distribution of the main academic CAR-T initiatives across three Brazilian states. (Right) Main institutions and their roles in each state. São Paulo concentrates academic manufacturing and clinical development, spanning São Paulo city (Hospital Israelita Albert Einstein, USP-FMUSP, Butantan Institute) and Ribeirão Preto (Ribeirão Preto Blood Center-FMRP-USP, Nutera-Ribeirão Preto). Rio de Janeiro concentrates vector production and translational research, hosting the National Cancer Institute (INCA) and Fiocruz/Bio-Manguinhos. Ceará hosts antibody engineering and preclinical CAR development at Fiocruz Ceará (GEPeSS, Protein Engineering and Health Solutions Group). Created in BioRender. LNK, L. (2026) https://BioRender.com/j877ab3 (agreement no. MA2A6W3C2N).

The CARTHIAE (NCT05705570) trial, conducted at Hospital Israelita Albert Einstein and funded by the Brazilian Ministry of Health through Institutional Development Support Program of the Unified Health System (PROADI-SUS), evaluates an autologous anti-CD19 CAR-T product for relapsed/refractory CD19-positive B cell lymphoid malignancies (acute lymphoblastic leukemia, chronic lymphocytic leukemia, and diffuse large B cell lymphoma). The trial employs a hospital-based point-of-care manufacturing strategy using an automated, closed platform, enabling the complete manufacturing process to be performed within a single institution in approximately 12 days. Preliminary results reported an overall response rate of approximately 81% and a complete remission rate of approximately 72%, with a toxicity profile comparable to that observed with internationally approved CAR-T products.^9^ This study represents an important milestone toward establishing locally manufactured CAR-T therapies within the Brazilian healthcare system.^4^^,^^9^^,^^10^

A parallel academic program has been developed since 2019 by the Ribeirão Preto Blood Center, USP, and Butantan Institute, formalized in 2022 through the Advanced Therapy Center (Nutera), with manufacturing units in Ribeirão Preto and São Paulo. This collaboration produces an anti-CD19 CAR-T product for relapsed/refractory B cell acute lymphoblastic leukemia and non-Hodgkin lymphoma, evaluated in the CARTHEDRALL (NCT06101381) phase 1/2 trial (funded by the Ministry of Health through PROCIS, a Ministry of Health program that supports technology transfer, national production, and innovation in health technologies), targeting patients across five centers.^11^ In December 2024, the Brazilian Health Regulatory Agency (ANVISA) approved the phase 1 safety report and authorized progression to phase 2.^11^ Preliminary results from this ongoing trial, presented by the Ministry of Health in June 2026, reported an overall response rate of 87.5% among the first 25 patients treated, all of whom had relapsed or refractory disease following prior chemotherapy, radiotherapy, or transplantation.^14^

Despite these advances, the Brazilian CAR-T ecosystem remains geographically concentrated. Most manufacturing facilities, research centers, and clinical trials are located in the state of São Paulo, with additional established initiatives concentrated in Rio de Janeiro and emerging in Ceará (Figure 1). This uneven distribution reflects the concentration of specialized infrastructure, GMP facilities, experienced multidisciplinary teams, and investment in a limited number of institutions. Notably, the country’s major reference hospital centers for hematology and oncology are also concentrated in this same region, reinforcing rather than offsetting this geographic imbalance. Expanding manufacturing capacity to other regions through collaborative networks, technology-transfer programs, and decentralized manufacturing models will be essential to improve equitable patient access across the country.^5^^,^^6^^,^^7^^,^^8^

The Brazilian experience also highlights the challenges that remain before CAR-T therapy can be broadly implemented nationwide. Although commercial CAR-T products are currently available in Brazil, they remain entirely dependent on importation, resulting in high treatment costs, long manufacturing timelines, and limited accessibility.^15^ In parallel, academic programs continue to face obstacles related to GMP viral vector production, sustainable funding, workforce development, regulatory coordination, and future incorporation into the Brazilian Unified Health System (SUS).^11^ Nevertheless, the establishment of multiple complementary academic initiatives demonstrates that Brazil is progressively transitioning from technology adoption toward the development of an independent national CAR-T manufacturing ecosystem capable of supporting innovation and future clinical expansion.^5^^,^^6^^,^^7^^,^^8^

The following sections discuss the major components required to consolidate this ecosystem, including regulatory pathways, manufacturing technologies, QC, financing strategies, and future opportunities to expand patient access to CAR-T therapy in Brazil.

## Regulatory pathway for CAR-T therapy in Brazil

Unlike conventional pharmaceuticals, CAR-T products are classified as advanced therapy medicinal products (ATMPs), requiring a multidisciplinary regulatory framework involving multiple governmental agencies throughout product development from discovery phase through nonclinical and clinical development and, ultimately, to market authorization.^16^

### Regulatory framework during the discovery and nonclinical development phases

In Brazil, the development of genetically modified cellular products is subject to biosafety and ethical oversight from the preclinical stage. Studies involving genetically modified organisms (GMOs) must comply with the biosafety framework established by the National Technical Commission on Biosafety (CTNBio), including the applicable institutional biosafety requirements, independently of whether the product is subsequently advanced to clinical testing. Thus, CTNBio oversight is part of the preclinical development pathway for CAR-T candidates and is not a regulatory step that occurs only after completion of preclinical studies.

In parallel, preclinical research involving human-derived biological material, such as T cells obtained from healthy donors or patients, is subject to the regulations of the National Research Ethics Authority (INAEP), governed by law no. 14.874/2024, and all research projects must receive prior approval from the relevant institutional research ethics committees (CEPs).^17^ Appropriate ethical approval must be obtained before the collection and/or use of human biological material for research, in accordance with the applicable regulations for research involving human participants and human biological material. For studies involving stored human biological material, the relevant requirements for biobanks and biorepositories must also be observed.

In addition to biosafety and human research ethics requirements, nonclinical studies involving animals are governed by law no. 11.794/2008, which established the National Council for the Control of Animal Experimentation (CONCEA) as the national authority responsible for issuing standards on the ethical care and use of animals in teaching and scientific research.^18^^,^^19^ Institutions that breed, maintain, or use animals for research purposes must hold an ethics committee on animal use (CEUA), which is responsible for reviewing all projects involving animal experimentation. Every animal experimental protocol, including xenograft efficacy models and dose finding, pharmacokinetics, pharmacological safety, and toxicology nonclinical studies of CAR-T candidates, requires prior CEUA approval.

### Regulatory framework during clinical development phase

Once sufficient preclinical proof-of-concept and safety data support progression to human testing, the regulatory pathway advances to clinical investigation. At this stage, clinical protocols involving human participants require ethical approval through the applicable CEP and regulatory authorization from ANVISA. For investigational ATMPs, including CAR-T products, clinical trials are regulated primarily by Collegiate Board Resolution (RDC) no. 506/2021, which establishes the regulatory requirements and procedures for conducting clinical trials with investigational advanced therapy products in Brazil.^16^ The regulation applies to any clinical trial involving ATMPs, regardless of whether it is intended to support a marketing authorization, provided that it is aimed at generating safety and/or efficacy evidence.^16^

For class II ATMPs, which include extensively manipulated advanced cellular therapy products, including CAR-T products, the clinical development package is submitted to ANVISA through the Investigational Advanced Therapy Product Clinical Development Dossier (DDCTA). The DDCTA comprises the documentation required for ANVISA’s assessment of the proposed clinical development and includes, among other elements, the investigator’s brochure, the investigational product manufacturing dossier (chemical, manufacturing, and controls [CMC]), relevant nonclinical evidence, and the study-specific clinical trial documentation.^16^ The manufacturing dossier provides information on the facilities and processes involved in production, starting materials and raw materials, manufacturing procedures, characterization and QC of the active component and final product, analytical methods, stability data, and the critical nonclinical evidence supporting the proposed clinical development.^16^

The regulatory framework extends beyond the initial authorization of the clinical trial. During clinical development, sponsors are required to maintain regulatory oversight of the study through mechanisms including the reporting of safety information, annual clinical trial follow-up reports, substantial and non-substantial modifications to the DDCTA, and procedures for the suspension or termination of clinical trials when applicable.^16^ These requirements are particularly relevant to CAR-T products because their biological activity, persistence, and potential for delayed adverse events may require prolonged safety surveillance. ANVISA’s regulatory framework consequently establishes continued oversight throughout clinical development rather than treating authorization as a single point-in-time decision.

Manufacturing requirements are addressed in parallel through ANVISA’s GMP framework. Normative Instruction (IN) no. 270/2023, which consists of the adoption by ANVISA of Annex 2A of the PIC/S GMP Guide, establishes the GMP requirements specifically for ATMPs and applies to investigational and commercial products. It addresses the particular manufacturing characteristics of ATMPs and complements the broader GMP requirements applicable to medicinal products.^20^ Thus, for CAR-T clinical development, regulatory authorization depends not only on the adequacy of the clinical and nonclinical evidence contained in the DDCTA but also on appropriate control of the manufacturing process and product quality.

This regulatory burden is particularly challenging for academic and point-of-care developers, which often face substantial resource and infrastructure constraints compared with industry-sponsored development programs. Unlike jurisdictions with a formal “hospital-exemption” pathway for non-routinely manufactured ATMPs, such as the one used for the CAR-T product ARI-0001 in Spain under European Union (EU) regulation,^21^ Brazil currently requires academic and hospital-based CAR-T programs to pursue the same route as commercially developed products, regardless of whether the product is manufactured and administered within a single institution. As a result, academic developers face the same long-term pharmacovigilance and follow-up obligations described above as multinational sponsors, without an abbreviated regulatory track tailored to small-scale, non-routine, institution-based manufacturing, a structural difference from the European model that may be disproportionately harder to sustain for a single academic institution than for a commercial developer with dedicated regulatory affairs infrastructure and that may slow the translation of Brazilian manufacturing initiatives into approved clinical practice.^22^

This gap may be partially mitigated by ongoing efforts to strengthen early regulatory interaction with ANVISA. Through the Parlatório, the agency’s formal mechanism for regulatory dialogue, developers may seek clarification on regulatory requirements and product development considerations before formal submission of the DDCTA. However, these interactions do not currently constitute a dedicated and formally established pre-submission pathway specifically tailored to advanced therapy products, limiting the extent to which early regulatory dialogue can be considered a structured component of the CAR-T development pathway.

Taken together, the Brazilian regulatory framework establishes comprehensive oversight across the entire CAR-T development pathway, encompassing biosafety, ethical review, clinical investigation, manufacturing, product quality, and long-term safety monitoring. While this framework is essential to ensure the safety, quality, and clinical reliability of genetically modified cellular products, its complexity can represent a substantial barrier for academic and hospital-based developers. As Brazil seeks to expand national capacity for CAR-T development and manufacturing, an important regulatory challenge will therefore be to maintain rigorous standards of product quality and patient safety while creating regulatory pathways and institutional capabilities that are proportionate to the characteristics and risks of academic, non-commercial, and locally manufactured products. Strengthening regulatory expertise within academic centers, improving early interaction with ANVISA, and developing greater harmonization between regulatory, biosafety, ethical, and manufacturing requirements may be important steps toward enabling the translation of Brazilian CAR-T platforms from preclinical development to clinical application.

## Manufacturing challenges and technological autonomy in Brazil

The organizational model used for CAR-T production has important implications for treatment accessibility, logistics, and institutional autonomy.^23^ CAR-T centers can operate with different degrees of manufacturing independence: (1) user centers rely predominantly on externally manufactured products, with cell collection performed locally but engineering, expansion, QC, and product release conducted by an external manufacturer; (2) producer centers have greater manufacturing autonomy and perform major manufacturing steps on site, including activation, transduction, expansion, QC, and product release, while remaining dependent on externally supplied components such as clinical-grade viral vectors; and (3) developer centers represent the highest level of technological autonomy, integrating vector development and production with cell engineering, expansion, QC, and product release within the same institutional ecosystem.

In Brazil, the choice of CAR-T manufacturing strategy extends beyond technical considerations and is closely tied to a broader bottleneck in GMP-grade viral vector supply. Lentiviral vector manufacturing capacity remains limited worldwide, with substantial and variable lead times and high costs reported for GMP-grade products, including among well-funded commercial developers.^24^ For academic programs in Brazil, this dependence has historically translated into reliance on imported vectors, increasing both cost and manufacturing timelines and potentially limiting the number of patients that can be treated independently of clinical demand. An additional contributor to this supply-chain burden is the plasmid DNA required for viral vector production. Plasmids, as well as the cell lines used in vector manufacturing, constitute critical starting materials and must be appropriately manufactured, characterized, and controlled for their intended clinical use. The development of small-scale, fully disposable GMP manufacturing platforms has been proposed as one strategy to reduce the infrastructure and operational burden associated with plasmid production, particularly for early-phase academic programs.^25^

Two national initiatives illustrate different approaches to reducing dependence on imported viral vectors. First, Fiocruz, through its Bio-Manguinhos unit, signed a technology-transfer agreement with the non-profit organization Caring Cross in 2024 to establish domestic lentiviral and adeno-associated virus (AAV) vector production, using cleanroom infrastructure previously employed for COVID-19 vaccine manufacturing.^12^ Second, the Nutera network, established by Butantan Institute and the Ribeirão Preto Blood Center, is expanding national capacity for academic cell and viral vector manufacturing.^11^ Together, these initiatives suggest that Brazil is beginning to move beyond predominantly single-institution, point-of-care manufacturing toward more distributed production models. However, both approaches remain at an early stage of implementation, and publicly available data on production volumes, cost per dose, manufacturing timelines, and the extent to which domestic vector production can reduce costs and lead times compared with imported vectors remain limited.

Non-viral gene-delivery platforms offer a complementary strategy for reducing dependence on imported viral vectors. Transposon-based systems, mRNA electroporation, and lipid nanoparticle (LNP)-mediated delivery may offer simpler manufacturing workflows, lower production costs, and reduced reliance on GMP-grade viral vector supply chains.^26^^,^^27^^,^^28^ However, these approaches do not eliminate the need for appropriately qualified clinical-grade manufacturing inputs. Transposon-based systems, for example, still require appropriately manufactured and characterized plasmid DNA or other genetic components suitable for clinical use. Thus, although non-viral approaches can reduce dependence on viral vector manufacturing, they do not necessarily eliminate the need for controlled, quality-assured genetic delivery materials. These platforms also present important trade-offs. Many non-viral approaches currently achieve lower or less durable gene-transfer efficiency than established viral vectors, while transposon-based systems require additional characterization of genomic integration and insertional mutagenesis risks. By contrast, transient approaches such as mRNA electroporation avoid permanent genomic integration but may require repeated or otherwise optimized delivery strategies because of the transient nature of transgene expression.

Although CAR-T development in Brazil has so far relied predominantly on viral platforms, non-viral approaches are also being explored. Researchers at INCA have reported a preclinical Sleeping Beauty transposon-based anti-CD19 CAR-T protocol demonstrating antitumor activity *in vitro* and in an NOD scid gamma (NSG) xenograft model.^29^ Separately, a piggyBac transposon-based anti-HER2 CAR-T construct was presented at an internal Fiocruz symposium in 2022, although this work has not yet been published in a peer-reviewed journal.^30^ Both efforts remain at the preclinical stage, underscoring that, while non-viral platforms may provide a lower-cost alternative to viral vector-based manufacturing, they have not yet reached the clinical validation achieved by viral-vector-based CAR-T programs in Brazil.

Domestic viral vector production and non-viral gene-delivery technologies should therefore not necessarily be viewed as competing strategies. Rather, they may represent complementary routes toward greater technological autonomy. Establishing national GMP-grade plasmids and viral vector production could strengthen the manufacturing of clinically established CAR-T platforms and reduce dependence on imported vectors, while transposon-based and other non-viral approaches could reduce reliance on complex viral manufacturing workflows and provide alternative platforms for scalable academic production. The relative value of these approaches will ultimately depend on their reproducibility, manufacturing scalability, genomic safety, regulatory acceptance, and clinical validation, as well as their ability to generate meaningful reductions in manufacturing costs and improve patient access.

Manufacturing infrastructure alone, however, is insufficient to establish sustainable technological autonomy. Workforce constraints represent an additional and potentially underappreciated bottleneck. International analyses of the cell and gene therapy sector, including initiatives involving UK and US stakeholders, have identified shortages of appropriately trained GMP manufacturing personnel as a major operational constraint, with academic manufacturing sites particularly affected.^31^ Brazil has not yet published a national workforce census specific to cell and gene therapy manufacturing, making the magnitude of this gap difficult to quantify. This represents an important evidence gap, particularly because developing personnel with the specialized expertise required for GMP manufacturing may take substantially longer than establishing the physical infrastructure itself. Brazil’s experience with COVID-19 vaccine manufacturing provides one potential model for addressing this challenge: GMP-trained personnel from previous biotechnology programs were redeployed to support the establishment and operation of the Bio-Manguinhos/Fiocruz vaccine manufacturing infrastructure, illustrating how expertise developed in adjacent biopharmaceutical manufacturing activities can contribute to the expansion of national manufacturing capacity.^32^ Similar training pipelines could potentially be developed through viral vector production, biopharmaceutical manufacturing, and other advanced cell-processing activities to build a specialized workforce for CAR-T manufacturing.

Taken together, achieving genuine technological autonomy in Brazil will require more than replicating the point-of-care model at additional institutions. It will depend on the simultaneous development of reliable national supply chains for critical starting materials and viral vectors, scalable manufacturing infrastructure, standardized quality systems, and a sufficiently trained GMP workforce. The ability of national vector manufacturing initiatives to supply multiple clinical centers, the potential replication of models such as Nutera beyond São Paulo, and the expansion of specialized manufacturing expertise are therefore interdependent components of the same technological ecosystem. At present, these dimensions are largely considered separately in the literature, and their combined feasibility and economic impact in Brazil remain insufficiently characterized.

## Framework for CAR-T therapy implementation in Brazil

In Brazil, the clinical logistics of CAR-T cell therapy require not only an integrated infrastructure between manufacturing facilities and treatment centers but also adaptation to local regulatory, geographic, and resource constraints. Likewise, the operational flow involves several critical steps: (1) patient screening and eligibility assessment, (2) leukapheresis scheduling, (3) controlled transportation of the starting material, (4) centralized manufacturing and product release, and (5) shipping of the final cell-therapy product.^16^ Each step demands validated procedures, packaging compliant with ANVISA standards, and direct communication channels between the hospital and production facility to track batch status and delivery times.

While the clinical and logistical framework is essential for CAR-T therapy implementation in Brazil, the feasibility and safety of this complex operational flow ultimately depend on rigorous QC and strict regulatory oversight throughout the manufacturing process.

## QC and regulatory aspects

The manufacture of genetically modified CAR-T cells involves a multistep process susceptible to variability and manufacturing failure: an extensive real-world European Society for Blood and Marrow Transplantation (EBMT) survey reported that nearly 4% of CAR-T manufacturing attempts failed and a further 3.8% of products were out of specification (OOS), predominantly due to inadequate viability, purity, or potency.^33^ These findings underscore the importance of a comprehensive control strategy and predefined specifications to ensure that the final product meets appropriate standards of identity, safety, purity, viability, and potency before clinical administration.

Internationally, the quality framework for CAR-T products is based on principles established by the International Council for Harmonisation (ICH) and complemented by specific guidance issued by regulatory authorities such as the US Food and Drug Administration (FDA) and the European Medicines Agency (EMA).^34^^,^^35^^,^^36^^,^^37^^,^^38^^,^^39^ These frameworks emphasize a lifecycle- and risk-based approach to product development, in which critical quality attributes (CQAs), critical process parameters, analytical procedures, specifications, and manufacturing controls are established on the basis of product and process knowledge.^34^^,^^35^^,^^36^^,^^37^ EMA guidance specifically addressing medicinal products containing genetically modified cells, including CAR-T cells and genome-edited products, emphasizes characterization of starting materials, the genetic modification process, manufacturing consistency, product characterization, potency, and safety.^38^^,^^39^

Within the Brazilian regulatory framework, QC requirements for ATMPs extend from the qualification and control of raw and starting materials to characterization of the active substance and release testing of the final cellular product. These activities are supported by requirements for validated analytical methods, in accordance with RDC no. 166/2017, and by GMP principles applicable throughout the manufacturing process. Thus, QC should be understood as an integrated component of the overall control strategy rather than as a set of tests performed solely at the final release stage.

Consistent with ICH, FDA, and EMA principles, the control strategy for CAR-T products should be product specific and should be established according to the product’s CQAs, mechanism of action, manufacturing process, and associated risks, following a quality-by-design (QbD) approach. Consequently, there is no universal panel of assays or acceptance criteria applicable to all CAR-T products. Rather, specifications and analytical methods should be scientifically justified and established based on the characteristics and intended use of each product. Nevertheless, CAR-T product characterization and release testing generally require key attributes related to safety, identity, purity, and potency.

Safety testing includes sterility, endotoxin, mycoplasma, replication-competent lentivirus/retrovirus (RCL/RCR), and vector copy number (VCN). Identity assessment generally includes cell viability and CAR expression by flow cytometry, whereas purity encompasses the proportion and phenotype of the intended cellular population as well as relevant process- and product-related impurities. Potency assays should reflect the product-specific mechanism of action and may include cytotoxicity, cytokine production, degranulation, and functional phenotype assays.^40^^,^^41^^,^^42^^,^^43^^,^^44^^,^^45^^,^^46^^,^^47^^,^^48^^,^^49^ For products manufactured using integrating vectors, VCN is an important safety attribute and should be monitored according to product-specific specifications and applicable regulatory guidance. Products generated using genome-editing technologies additionally require assessment of unintended genomic modifications, including off-target editing and chromosomal abnormalities.^43^

Analytical procedures used for characterization and release should be demonstrated to be fit for their intended purpose and appropriately validated or qualified according to the stage of product development. Consistent with internationally accepted analytical-development principles, assay performance characteristics and acceptance criteria should be scientifically justified according to the attribute being measured, the analytical technology employed, and the intended use of the method.^36^^,^^37^

Taken together, these quality attributes should be interpreted as components of an integrated, product-specific release strategy rather than as isolated release assays. A QbD-based approach enables the identification of CQAs and the establishment of appropriate analytical methods and acceptance criteria based on a systematic understanding of the relationship between process parameters, product characteristics, and clinical performance. As Brazilian academic CAR-T programs move toward decentralized and point-of-care manufacturing, harmonization of analytical methods, assay validation, release specifications, and comparability strategies across manufacturing sites will become increasingly important to ensure consistent product quality, process control, and patient safety. Alignment with internationally accepted ICH, FDA, and EMA principles will be essential to ensure manufacturing consistency, comparability between sites, robust process control, and patient safety.

## Clinical implementation and patient management in Brazil

Successful CAR-T implementation depends on coordinated clinical pathways encompassing patient selection and preparation, leukapheresis, manufacturing, lymphodepletion, infusion, toxicity monitoring, and long-term follow-up. The CAR-T treatment pathway generally includes a pre-leukapheresis period aimed at optimizing disease control and T cell fitness, bridging therapy when required to control disease during manufacturing, lymphodepletion, CAR-T cell infusion, and intensive monitoring during the period of highest risk for acute toxicities.^50^^,^^51^^,^^52^ Lymphodepletion, most commonly based on fludarabine and cyclophosphamide, is administered before CAR-T infusion to promote an immunological environment favorable to CAR-T expansion and persistence.^50^

In Brazil, clinical implementation is influenced by the limited number of centers with extensive experience in CAR-T therapy and by the specialized infrastructure and multidisciplinary expertise required for the management of potentially severe treatment-related toxicities. Consequently, close monitoring following infusion remains particularly important. CAR-T treatment centers must have immediate access to supportive therapies, including tocilizumab for the management of cytokine release syndrome (CRS), corticosteroids when clinically indicated, and clearly established protocols for recognition and escalation of patients with severe CRS, immune effector cell-associated neurotoxicity syndrome (ICANS), and other complications requiring intensive care.^50^ Although outpatient CAR-T administration and monitoring models have increasingly been implemented in selected North American and European centers, these approaches are not yet widely adopted in Brazil, where logistical, infrastructural, regulatory, and patient-safety considerations may limit their implementation.^53^^,^^54^

Long-term follow-up represents another critical component of CAR-T implementation. Post-infusion monitoring incorporates recommendations from international organizations, including the EBMT and the American Society for Transplantation and Cellular Therapy (ASTCT), and includes surveillance for delayed toxicities, infections, immune reconstitution, secondary malignancies, and other treatment-related complications.^55^ In Brazil, long-term follow-up may be particularly challenging because specialized CAR-T centers are geographically concentrated, requiring patients from distant regions to travel long distances or transition their care to local healthcare services. Effective coordination between CAR-T centers and regional healthcare networks will therefore be essential to ensure continuity of care, including infection prophylaxis, vaccination, monitoring of immune reconstitution, management of late complications, and long-term clinical surveillance.

The complexity of CAR-T therapy also requires a highly integrated multidisciplinary team spanning the entire treatment pathway. Hematologists and oncologists, cell-therapy specialists, apheresis and cellular processing teams, pharmacists, nurses, infectious disease specialists, intensivists, neurologists, laboratory and quality professionals, and other specialized staff must work in close coordination to ensure appropriate patient selection, product handling, timely recognition, management of toxicities, and long-term follow-up. This multidisciplinary infrastructure is particularly important in Brazil, where CAR-T therapy is currently available at a limited number of specialized centers, while access to trained personnel and critical care resources varies substantially across regions. Strengthening multidisciplinary teams, standardized training programs, referral, and collaboration between specialized CAR-T centers and regional healthcare services will therefore be essential for expanding access while maintaining the safety and quality of CAR-T therapy as its use increases in the country.

## Financing, reimbursement, and integration into the Brazilian SUS

The cost of a single commercial CAR-T treatment has been reported to range from approximately USD 373,000 to 475,000, excluding additional expenditures related to hospitalization, supportive care, and management of treatment-associated toxicities.^56^ These costs reflect not only the individualized manufacturing process but also the requirement for specialized infrastructure, highly trained personnel, rigorous QC, logistics, and prolonged patient monitoring. Importantly, the economic burden of CAR-T therapy extends beyond the price of the cellular product, encompassing the complete treatment episode from leukapheresis and manufacturing through lymphodepletion, infusion, toxicity management, and long-term follow-up.

In this context, the development of academic and locally manufactured CAR-T platforms represents an important strategy for investigating whether greater national manufacturing capacity can reduce dependence on imported commercial products and improve the economic sustainability of these therapies. National production may also reduce exposure to international supply chains, transportation costs, and currency fluctuations while strengthening domestic technological capabilities. However, lower manufacturing costs alone do not guarantee sustainable patient access, as CAR-T therapy requires substantial investment in infrastructure, quality systems, hospitalization capacity, toxicity management, specialized multidisciplinary teams, and long-term patient follow-up. The economic value of national manufacturing should therefore be evaluated in terms of its potential impact on the total cost of care rather than manufacturing costs alone.

In Brazil, several public funding mechanisms have supported academic research and the establishment of national CAR-T manufacturing capabilities. Programs such as PROADI-SUS and National Program to Support Oncology Care (PRONON) provide strategic resources for translational and clinical research in oncology.^57^ National and state funding agencies, including São Paulo Research Foundation (FAPESP), National Council for Scientific and Technological Development (CNPq), Funding Authority for Studies and Projects (FINEP), and Brazilian Company of Research and Industrial Innovation (EMBRAPII), have also contributed to strengthening national biomanufacturing capabilities and fostering innovation in cell and gene therapies. In 2023, the Brazilian Ministry of Health allocated R$205.2 million through programs including PRONON and PROADI-SUS to support research activities across 12 institutions.^58^ Philanthropic organizations and public-private partnerships may provide complementary mechanisms for supporting infrastructure development, technology transfer, and clinical translation.

Nevertheless, research funding and manufacturing capacity represent only part of the pathway toward broad patient access. In the private healthcare sector, CAR-T treatment requires coordination between health insurance operators, hospitals, specialized cell-therapy centers, and manufacturers or academic production facilities. Unlike conventional medicines, CAR-T therapy combines a highly specialized cellular product with a complex sequence of healthcare services. Consequently, coverage and payment cannot be considered solely in terms of the acquisition of the cellular product, and the allocation of costs among the different components of the treatment episode may represent an important challenge. The Brazilian National Regulatory Agency for Private Health Insurance and Plans (ANS) establishes the list of health procedures and events, which defines the minimum mandatory coverage for regulated health plans.^59^ For advanced therapies such as CAR-T, however, the interaction between regulatory coverage, contractual provisions, clinical indication, and the specialized infrastructure required for treatment may create additional complexity in establishing clear payment pathways.

Within the Brazilian SUS, the challenge is broader than reimbursement alone and involves the incorporation, financing, and delivery of CAR-T therapy within the public healthcare network. The incorporation of high-cost technologies requires assessment of clinical effectiveness, safety, cost-effectiveness, and budget impact, while also considering the infrastructure and specialized services required for their implementation.^60^ Moreover, the geographic concentration of specialized CAR-T centers in Brazil may create additional barriers to equitable access, particularly for patients living far from reference institutions. Therefore, integration into SUS will require not only sustainable financing but also coordinated referral networks and collaboration between specialized CAR-T centers.

These challenges also highlight the need to distinguish the price of CAR-T manufacturing from the total cost of care. A locally manufactured product may have a substantially lower acquisition cost than an imported commercial therapy, but the overall economic benefit will depend on manufacturing scalability, product quality and consistency, clinical outcomes, hospitalization requirements, toxicity rates, and long-term healthcare utilization. Prospective health-economic studies using Brazilian clinical and healthcare-resource data will therefore be important to determine whether locally developed CAR-T platforms can provide comparable clinical benefit at a lower overall cost to the healthcare system.

Alternative financing and payment models may also become relevant as CAR-T therapy expands in Brazil. Outcomes-based agreements, risk-sharing arrangements, staged payments, and other value-based approaches could help address the high upfront expenditure associated with these therapies by distributing financial risk between payers and manufacturers or linking part of the payment to predefined clinical outcomes.^61^ Although implementation of such models would require robust longitudinal follow-up and standardized outcome measures, they may offer a potential framework for reconciling the high initial cost of CAR-T therapy with the possibility of durable clinical benefit after a single treatment episode.

Ultimately, the successful integration of CAR-T therapy into the Brazilian healthcare system will depend on aligning national manufacturing capacity with appropriate health technology assessment, sustainable financing mechanisms, clinical infrastructure, and equitable access. The development of locally manufactured products may contribute to technological autonomy and potentially reduce treatment costs, but these benefits will only translate into broader access if the healthcare system is able to finance and deliver the complete treatment pathway. In this regard, CAR-T should be considered not simply as a high-cost cellular product but as a complex healthcare intervention requiring coordinated investment in manufacturing, clinical care, infrastructure, and long-term patient management.

## Future perspectives: Expanding access through next-generation CAR platforms

The next phase of CAR-based therapy development in Brazil will likely extend beyond optimization of conventional autologous CAR-T manufacturing toward more scalable cellular platforms. In contrast to individualized autologous approaches, allogeneic CAR-based therapies may enable the development of standardized, off-the-shelf products, potentially reducing manufacturing timelines, simplifying logistics, and allowing a single manufacturing campaign to generate therapeutic doses for multiple patients. Potential cellular sources include healthy donor-derived T cells, umbilical cord blood, induced pluripotent stem cells (iPSCs), and alternative immune effectors such as natural killer (NK) cells.^62^ These approaches may be particularly relevant in Brazil, where manufacturing capacity, geographic distribution of specialized centers, and treatment costs remain important barriers to broader access.

Brazilian preclinical research is increasingly exploring these alternative platforms, including allogeneic CAR-T and CAR-NK approaches using different cellular sources and strategies to improve product scalability and standardization. In this emerging landscape, Hospital Israelita Albert Einstein has established initiatives, supported by the Brazilian PRONON, investigating CAR-NK platforms for both hematological and solid tumors, as well as allogeneic CAR-T strategies incorporating base-editing approaches to enable targeted genome engineering. Preliminary results from these studies are encouraging, although these programs remain under active development and further validation is required. These initiatives remain predominantly at the preclinical or early translational stage but represent an important opportunity to build national expertise in next-generation cellular engineering and manufacturing. Genome-editing technologies may further support the development of allogeneic products by reducing the risks of graft-versus-host disease and host-mediated immune rejection.^63^ However, these approaches also increase product complexity and introduce additional requirements for characterization, QC, genomic safety, and manufacturing consistency.

In parallel, *in vivo* CAR-T generation, using viral vectors or lipid nanoparticle (LNP)-delivered mRNA to directly program T cells within the patient’s body, has emerged as another strategy to bypass conventional *ex vivo* manufacturing, potentially reducing costs, manufacturing timelines, and infrastructure requirements.^64^ Although this approach remains at an early clinical stage internationally, research in Brazil is also beginning to advance this field.^65^

Notably, a group at the Federal University of Minas Gerais (UFMG) has reported the development of ionizable LNP platforms for T cell engineering, with published preclinical studies demonstrating CAR expression following plasmid DNA delivery.^66^ Building on these findings, the group is currently extending this platform to mRNA-based CAR engineering, an approach that could enable transient *in vivo* programming of T cells and further reduce the complexity associated with conventional *ex vivo* CAR-T manufacturing. These efforts highlight the potential of LNP-based approaches as an emerging strategy to simplify CAR-T manufacturing and expand access to advanced cellular therapies, particularly in resource-limited settings.

Beyond technological development, translation of these platforms will require continued strengthening and harmonization of regulatory, manufacturing, and analytical frameworks. Alignment of regulatory expectations, quality standards, analytical methods, potency assays, release criteria, and comparability strategies across academic institutions and clinical centers will become increasingly important as Brazilian programs progress toward clinical development. Strengthening shared infrastructure, technical expertise, standardized procedures, and early interaction with regulatory authorities could facilitate technology transfer and multicenter development without compromising product quality or patient safety.

## Conclusion

The development of CAR-T cell therapy in Brazil represents both a scientific milestone and a public health opportunity. Over the past decade, academic institutions have made meaningful progress in establishing national platforms capable of producing clinical-grade CAR-T products. Initiatives led by centers such as Hospital Israelita Albert Einstein, USP/Butantan, and Fiocruz illustrate the strategic value of fostering local innovation to reduce dependence on imported commercial products. However, these platforms remain at an early and geographically concentrated stage, and their translation into broad, equitable clinical access is still in development.

Several substantial challenges must be resolved before CAR-T therapies can be widely implemented across the Brazilian healthcare system. Establishing sustainable manufacturing pipelines will require not only domestic vector production capacity currently in early implementation at institutions such as Fiocruz but also a trained GMP workforce whose current size and growth rate are not yet documented in the literature. Regulatory coordination among CTNBio, ANVISA, and CEP/CONEP, while already structured, continues to demand harmonization as decentralized and point-of-care manufacturing models expand. Most critically, integrating these therapies into the SUS will require formal health technology assessment, demonstrated cost-effectiveness relative to imported products, and reimbursement mechanisms that do not yet exist, a gap that, if unaddressed, could limit the benefits of local manufacturing to a small number of specialized centers rather than the broader patient population.

The Brazilian experience illustrates the role academia can play in expanding access to advanced cellular therapies, provided that manufacturing gains are matched by parallel progress in workforce training, regulatory harmonization, and reimbursement policy. Continued collaboration among research institutions, government agencies, and industry partners may allow Brazil to reduce its dependence on imported technologies. Whether this translates into a model transferable to other low- and middle-income countries will depend on evidence not yet available: comparative cost-effectiveness data, real-world manufacturing throughput beyond pilot studies, and documented expansion of production capacity outside São Paulo. Reporting these outcomes as they emerge, rather than assuming them, will be essential for the Brazilian CAR-T ecosystem to move from proof of concept to sustained, nationwide clinical impact.

## Acknowledgments

The authors gratefully acknowledge colleagues and collaborators for their valuable scientific discussions and critical insights that contributed to the development of this review. We also acknowledge the institutional support provided by the affiliated research centers, which offered an intellectually stimulating environment conducive to the preparation of this manuscript. This work was primarily supported by the American Society of Hematology (ASH) through the ASH Global Research Award. Additional funding was provided by the PRONON (grant no. 25000.196663/2019-60; 25000.169325/2020-99; 25000.137130/2023-22). Some individual investigators contributing to this study were also supported by the FAPESP (grant nos. 2024/08168-2, 2025/04084-0, and 2024/18226-0). The Graphical Abstract is created in BioRender. LNK, L. (2026) https://BioRender.com/qhhzccr” (Agreement no. UC2A6W3I12).

## Author contributions

C.d.C.O.B. conceived the review, coordinated the writing process, and served as corresponding author. L.C.Z. revised the entire manuscript, provided scientific input, and contributed to writing several sections. L.A.M.T.-S., J.C.B., E.K.S.V., I.F.d.S., and R.A.F. performed the literature search and drafted sections of the manuscript. L.d.O.C., L.J.A., J.R.d.S., and Y.A. contributed to the critical analysis of the regulatory and manufacturing literature and revised the manuscript. R.d.M.A.P. and O.K.O. provided critical scientific input on cell and gene therapy manufacturing aspects and revised the manuscript for intellectual content. J.A.P.d.G. wrote and provided intellectual input for the QC section. A.B. revised and provided intellectual input on Brazilian regulation pathway. N.H. contributed to the clinical and translational perspective and critically reviewed the manuscript. R.C.V.P. provided critical scientific input on viral vector manufacturing (plasmid production, GMP workforce considerations) and revised the manuscript for intellectual content. L.N.K. and L.C.Z. supervised the study and critically revised the final version of the manuscript. All authors read and approved the final manuscript.

## Declaration of interests

The authors declare no competing interests.
